# Tumour infiltrating lymphocytes correlate with improved survival in patients with esophageal squamous cell carcinoma

**DOI:** 10.1038/srep44823

**Published:** 2017-03-21

**Authors:** Dongxian Jiang, Yalan Liu, Hao Wang, Haixing Wang, Qi Song, Akesu Sujie, Jie Huang, Yifan Xu, Haiying Zeng, Lijie Tan, Yingyong Hou, Chen Xu

**Affiliations:** 1 Department of Pathology, Zhongshan Hospital, Fudan University, Shanghai 200032, P. R. China; 2 Department of Thoracic surgery, Zhongshan Hospital, Fudan University, Shanghai 200032, P. R. China; 3 Department of Pathology, School of Basic Medical Sciences & Zhongshan Hospital, Fudan University, Shanghai 200032, P. R. China

## Abstract

We undertook a study of tumour infiltrating lymphocytes (TILs) in a large and relatively homogeneous group of patients with completely resected esophageal squamous cell carcinoma (ESCC). Hematoxylin and eosin–stained sections of 235 ESCC tumours were evaluated for density of TILs in intratumoural (iTIL) and stromal compartments (sTIL). Foxp3+, CD4+, and CD8+ T cells in tumoural and stromal areas were evaluated by immunohistochemistry. Of the 235 tumours, high sTIL (>10%), and iTIL (>10%) were observed in 101 (43.0%) and 98 (41.7%), respectively. The median follow-up period was 36.0 months (95% CI 29.929–42.071). Univariate analysis revealed that sTIL (>10%), iTIL (>20%), vessels involvement, lymph node metastasis, and clinical stage were significantly associated with postoperative outcome. In multivariate analysis, high sTIL (HR: 0.664, *P* = 0.019 for Disease free survival; HR: 0.608, *P* = 0.005 for Overall survival) was identified as independent better prognostic factor. Further analysis, sTIL was identified as independently prognostic factor in Stage III-IVa disease, which was not found in Stage I-II disease. Our study demonstrated that sTIL was associated with better ESCC patients’ survival, especially in Stage III-IVa disease. Assessment of sTIL could be useful to discriminate biological behavior for ESCC patients.

Esophageal carcinoma (EC) is one of the most prevalent life threatening malignancies^1^, which ranks as the eighth most frequently diagnosed cancer, and the sixth leading cause of cancer death worldwide^2^,^3^. China alone accounts for more than 70% of global EC, and 95% is esophageal squamous cell carcinoma (ESCC), with an estimated 478,000 new cases and 375,000 new deaths in 2015^4^,^5^. As the survival of ESCC remains poor, prognostic markers are required to discriminate the different biologic behavior of ESCC in order to make the following clinical strategies.

Since Virchow first described the association of lymphocyte infiltration with solid tumours in 1863^6^, tumour infiltrating lymphocytes (TILs) are increasingly involved in determining the progression and aggressiveness of tumour. TILs are composed of various lymphocytes with diverse activities. The most common lymphocytes are CD8+, CD4+, and Foxp3+ T cells. Of them, CD8+ T cells play a critical role in antitumour immunity by releasing perforins and granzymes, which may contribute to tumour cell death^7^. CD4+ T cells release immunoregulatory cytokines such as IFN-γ and TNF that may induce cytolytic T cell responses in tumours. Foxp3+ T cells have the ability to suppress or regulate cell-mediate immunity^8^. After infiltrating solid tumours, Foxp3+ T cells can inhibit the activity of cytotoxic T cells through cell-to-cell contact.

Emerging evidence suggests that the amount of T lymphocyte infiltration of primary tumours consistently predicts favorable outcomes in a number of tumour types, including breast cancer^9^, head and neck cancer^10^, non-small cell lung cancer^11^, colorectal cancer^12^ and gastric cancer^13^. However, tumour progression, which may occur in the presence of substantial lymphocytic infiltration, suggests that T cells are not capable of providing effective immune responses to control tumour growth in some types of cancer^14^. What’s more, regarding the infiltration of CD4+, CD8+, Foxp3+ T cells, there are also conflicting results. For example, some studies showed an association between increased numbers of CD8+ T cells and longer survival^10^,^15^,^16^, while others showed an association with shorter survival^17^,^18^.

A few initial studies examined prognostic implications of TILs found in ESCC. Schumacher *et al*. suggested the presence of TILs was associated with improved survival in a cohort of ESCC patients (n = 33)^19^. Yoshioka *et al*. found TILs were not associated with improved long-term survival, however^20^. Therefore, the prognostic value of TILs in ESCC remains to be established and the composition of TILs density in ESCC are yet to be assessed.

We undertook a study of TILs in a large and relatively homogeneous group of patients with completely resected ESCC to better determine correlations with clinical prognostic variables and to evaluate the overall impact of specific TILs populations on patient outcomes.

## Results

### Study patients

A total of 235 patients were included in the study. The median age at the time of surgery was 62 years (range, 37–83 years) and was predominately male (83.8%). Of all patients, 57.0% were non-smokers and 42.6% were smokers. On the basis of the AJCC Staging Manual (seventh edition), 4 (1.7%) cases were histologically graded as well differentiated, 133 (56.6%) cases moderately differentiated, and 98 (41.7%) poorly differentiated. Vessel and nerve invasion (once present with no matter of the quantification) were identified in 46 (19.6%) and 69 (29.4%) tumours, respectively. Lymph node metastasis was identified in 124 (52.8%) patients. Among these patients, 136 (57.9%) patients had AJCC pathologic stage I–II disease, and 99 (42.1%) stage III-IVa disease, which were located in the upper esophagus (5.1%), middle esophagus (47.7%) or lower esophagus (43.4%) (Table 1).

### The relationship of TILs and clinicopathological characteristics

**Figure 1:**
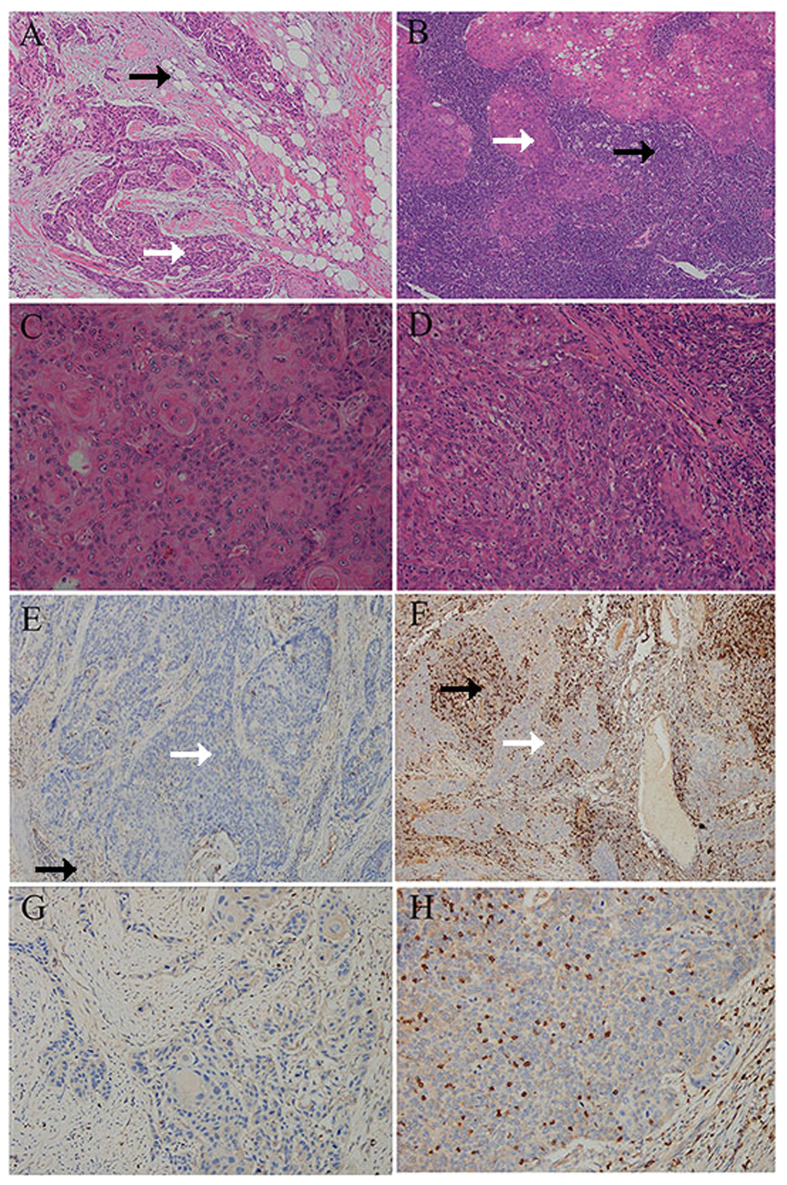
Representative views of tumour infiltrating lymphocytes (TILs) and CD8 expression in ESCC. Examples of low sTIL (**A**) and high sTIL (**B**) in stromal compartments (magnification 10×), low iTIL (**C**) and high iTIL (**D**) in intraepithelial compartments (magnification 40×), CD8+ low sTIL (**E**) and CD8+ high sTIL (**F**) in stromal compartments (magnification 10×), CD8+ low iTIL (**G**) and CD8+ high iTIL (**H**) in intraepithelial compartments (magnification 40×). White and black arrows indicate tumoural and stromal area respectively.

Representative TILs staining is shown in Fig. 1. Of the 235 tumours, high sTIL (>10%), iTIL (>10%) and iTIL (>20%) were observed in 101 (43.0%), 98 (41.7%), and 23 (9.8%) respectively. The relationship of patient and tumour characteristics to TILs involvement is presented in Table 1. High sTIL (>10%) density was associated with tumour site (*P* = 0.026), and high iTIL (>10%) was observed in tumours with poorer differentiation (*P* = 0.024). No statistically significant correlation was observed with sex, age, vessel and nerve involvement, lymph node metastasis, clinical stage, or smoking behavior (Table 1).

The median number of CD4+ sTIL and iTIL was 2% and 1%, respectively. The median number of CD8+ sTIL and iTIL was 5%, the median number of Foxp3+ sTIL and iTIL also was 5%. Among high sTIL (>10%) cases, 16 (15.8%) were high CD4+ sTIL (>10%), 58 (57.4%) were high CD8+ sTIL (>10%), and 3 (3.0%) were high Foxp3+ sTIL (>10%). Among high iTIL (>10%) cases, no cases were comprised of high CD4+ iTIL (>10%), 58 (59.2%) were high CD8+ iTIL (>10%), and 5 (5.1%) were high Foxp3+ iTIL (>10%). Both sTIL and iTIL were statistically associated with CD8+ cells (*P* < 0.05).

### Prognostic significance of TILs

The median follow-up period was 36.0 months (95% CI 29.929–42.071). There were 142 (60.4%) instances of tumour progression documented. Median DFS was 24.0 months. A total of 139 patients (59.1%) died of ESCC during the follow up, and median OS was 32.0 months.

To clarify whether TILs could have a prognostic value, univariate and multivariate survival analyses were performed in all cases. Our univariate analysis revealed that sTIL (>10%), iTIL (>20%), vessel involvement, lymph node metastasis and clinical stage were significantly associated with postoperative outcome as described in Table 2.

In the multivariate analysis, high sTIL (HR: 0.664, *P* = 0.019 for DFS; HR: 0.608, *P* = 0.005 for OS) was identified as independent better prognostic factor, and lymph node metastasis (HR: 1.950, *P* = 0.010 for DFS; HR: 2.118, *P* = 0.004 for OS) and clinical stage (HR: 2.040, *P* = 0.004 for DFS; HR: 2.119, *P* = 0.002 for OS) were identified as independent worse prognostic factors as shown in Table 2.

**Figure 2:**
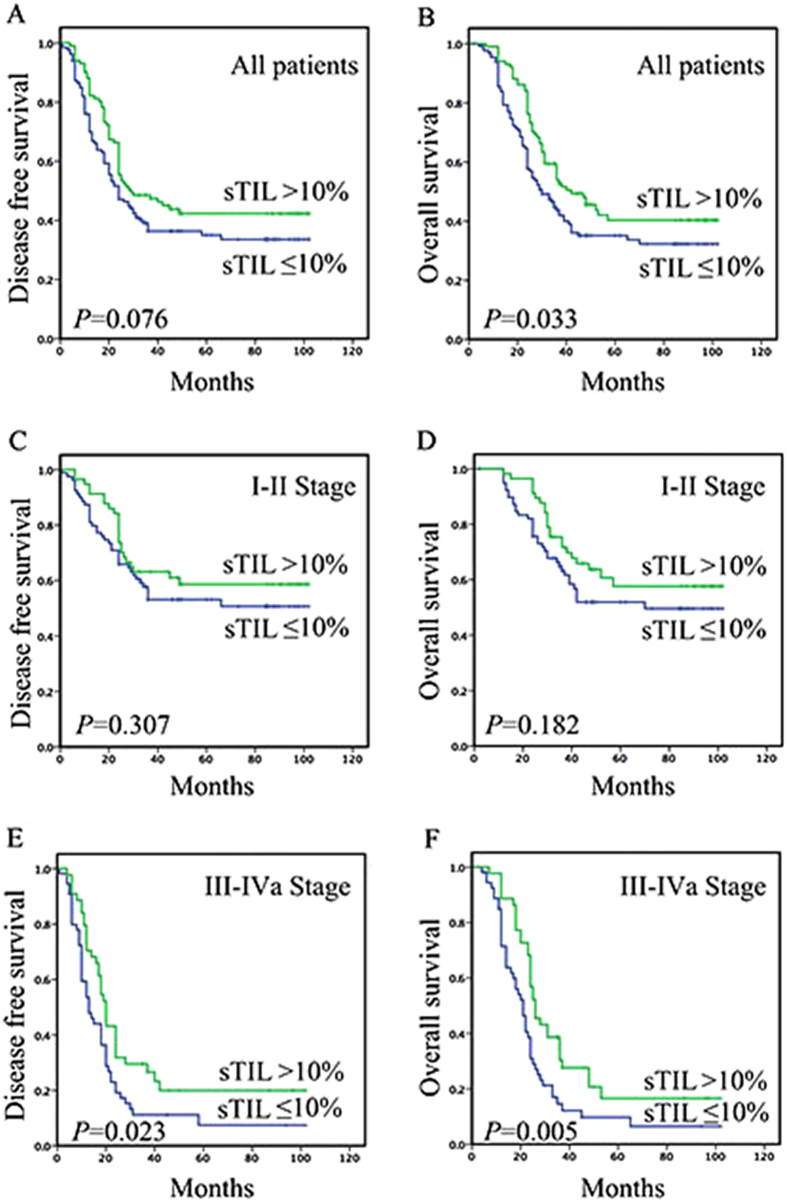
Kaplan–Meier curves of DFS and OS revealing prognostic significance of TILs in ESCC. Among 235 patients, a significantly better prognosis was observed in patients with high sTIL (>10%) (*P* = 0.076 and 0.033) (**A** and **B**). In patients with Stage I-II ESCC, sTIL (>10%) was not associated with DFS (*P* = 0.307) and OS (*P* = 0.182) (**C** and **D**). In patients with Stage III-IVa disease, sTIL (>10%) was associated with improved DFS (*P* = 0.023) and OS (*P* = 0.005) (**E** and **F**).

Representative Kaplan–Meier survival curves according to these factors are shown in Fig. 2. Among 101 patients with high sTIL (>10%), a significantly better prognosis was observed, with a median DFS and OS of 29.0 and 42.0 months compared to 24.0 and 30.0 months for 134 patients with low sTIL (≤10%) (*P* = 0.076 and 0.033).

### Survival analyses based on clinical stage

In patients with Stage III-IVa disease, sTIL (>10%) was associated with improved DFS (*P* = 0.023) and OS (*P* = 0.005) (Fig. 2). Among 44 patients with high sTIL (>10%), a significantly better prognosis was observed, with a median DFS and OS of 20.0 and 26.0 months compared to 13.0 and 21.0 months for 55 patients with low sTIL (≤10%). Upon univariate analysis, sTIL, iTIL (>20%), sCD8 (>10%) and site were associated with DFS and OS, and on multivariate analysis, sTIL and site were identified as independently prognostic factor (Table 3).

In patients with Stage I-II ESCC, sTIL (>10%) was not associated with DFS (*P* = 0.307) and OS (*P* = 0.182) (Fig. 2). Upon univariate and multivariate analysis, lymph node metastasis and site were associated with DFS and OS (Table 4).

## Discussion

The aim of our study was to examine the prognostic relevance of TILs involvement in patients with ESCC. We found sTIL appears to have a superior prognostic impact when compared with iTIL, which is previously reported in other tumours^21^,^22^. In this study, we confirmed that sTIL constitutes a robust and independent prognostic marker in ESCC, with increasing sTIL predictive of a significantly lower risk of recurrence or metastasis, and overall mortality. At a median follow-up of 36.0 months, sTIL was associated with OS, with an estimated HR of 0.697 (*P* = 0.037). Importantly, the prognostic significance of sTIL was independent of known prognostic factors, and in our population of I-II stage and III-IVa stage diseases, it improved outcome prediction even more significantly when clinical stage was taken into account.

Given the increasing evidence of the importance of TILs in influencing prognosis of patients with tumours, the International TILS Working Group recently published a guideline to standardize TILs evaluation^23^. The working group states that evaluating sTIL as a continuous variable will allow for more accurate statistical analysis, but in practice, most pathologists will not report specific values. Simplicity is needed for a pathological methodology to be accepted widely. Typically, TILs are reported to the nearest 10–15%^23^. And some studies recommended TILs should be defined as >50% TILs^24^. Our work suggests that evaluating sTIL as a noncontinuous variable can be considered. In the study, we used a cutoff value of 10% to define low and high TILs. Statistical analyses could not be performed using a cut-off of 50% as few patients had TILs percentages this high. At the median follow-up period of 36 months, OS analysis (log-rank) showed sTIL to be prognostically significant. This is consistent with work from Hida and Ohi^25^, who recently published a letter to the editor commenting that the methodology proposed by the TILs working group is too detailed for routine use. They proposed a simpler method whereby they quantified TILs as low, intermediate, or high using 10, 10 to 50% or more than 50%. In a series of 164 consecutive triple-negative breast cancer specimens, they showed that using such a three-grade scale for TILs quantification, they could stratify patients with respect to survival.

It is well recognized that cytotoxic CD4+, CD8+ and Foxp3+ TIL constitute the most important effector mechanisms of anti-tumour immunity. However, some studies have found CD4+, CD8+ and Foxp3+ TIL not to be a better, more reproducible prognostic parameter. For example, in NSCLC, CD8+ TIL had ever been identified as better, poor, or no prognostic role^26^,^27^. In esophageal cancer, Schumacher *et al*. observed CD8+ iTIL to correlate with improved survival^19^. In contrast, Zingg *et al*. did not find any independent associations between CD8+ TIL subsets and survival^28^. Tumours infiltrated with Foxp3+ T cells were reported to have a worse prognosis by some investigators^29^,^30^, but others indicated that tumours with Foxp3+ T cells had better prognosis or non-prognosis role^16^,^31^. It is true that the prognostic value of TIL subtyping continues to be debated. Therefore, whether these markers add value beyond general sTIL enumeration on H&E-stained slides has not been established. Indeed, the working group stated that outside the research setting, routine use of IHC to differentiate lymphocyte populations is not recommended^24^. In our study, associations of CD4+, CD8+ and Foxp3+ TIL and outcome were also observed, and did not reach statistical significance. We found the density of stromal and intratumoural CD8+ TIL was significantly higher than CD4+ and Foxp3+ TIL. We speculated CD8+ T lymphocytes has the potent of cytotoxic effects in ESCC. Further studies are needed to explore the prognostic significance of different subsets of TILs in ESCC.

We have included cancer patients with all four stages (Stage I, II, II and IVa) in this study. More importantly, our study is the first report on the prognostic influence of TILs in both earlier (I+II) and later (III-IVa) stage ESCC. Some researches have found that the prognostic significance of biomarkers may differ in patients at varing points in the disease^32^,^33^.

Previous researches indicated that TILs analysis is a better predictor of patient survival at earlier stages of disease. For example, Ruffini and Horne *et al*. observed that TILs were associated with a significant survival advantage in Stage I NSCLC^34^,^35^. However, In our study, the prognostic effect was not statistically different in earlier stage ESCC (DFS *P* = 0.307 and OS *P* = 0.182), as some researchers reported in early breast cancer^14^. Therefore, the impact of TILs on I+II stage ESCC remains to be established.

As to Stage III-IVa ESCC, sTIL (>10%) was associated with improved DFS (*P* = 0.023) and OS (*P* = 0.005). In Stage III NSCLC, Feng and Lee at al. found that the TILs was independently associated with a favorable prognosis^36^,^37^. It is generally known that the conventional factors are insufficient for understanding the biological nature of III-IVa disease. TIL, with the consideration of both tumour and host defense, may provide clinically informative prognostic biomarker. Although our findings require further validation, it supports the concept that immune modulation could be particularly beneficial in improving clinical outcome for Stage III-IVa ESCC.

The patient prognosis after complete resection for Stage III-IVa ESCC remains a significant concern; the 5-year OS rates range from 10% to 30%. Our findings provided the impetus for identifying patients (low TILs) who are at high risk of recurrence and exploring novel therapeutic approaches for improving patient survival. High and low TILs ESCC may each reflect a distinct tumour cell biology that likely has markedly different susceptibility to immunotherapy. The experimental evidence supporting this notion awaits further investigation.

In summary, to our knowledge, the study is the first investigation of the prognostic role of TILs in a large series of ESCC patients. Our study showed that high sTIL is an independent, favorable prognostic factor in patients with surgically resected ESCC, especially III-IVa patients. To develop immune targeting drugs in future, more knowledge regarding the heterogeneous functions, homing, and regulatory mechanisms of TILs is needed.

## Materials and Methods

### Patients and sample selection

In this retrospective study, 235 unselected ESCC patients who received curative resection without preoperative chemotherapy at the Department of Thoracic Surgery, Zhongshan Hospital affiliated to Fudan University, Shanghai, China, between 2007 and 2010 were included. Clinicopathological information of patients in this survey was collected from clinical records and pathology reports. The local Ethics Committee of Zhongshan Hospital approved the protocol of this study, which was conducted in accordance with the Declaration of Helsinki. Written informed consent was obtained from all patients.

### Tissue microarray construction

Tissue microarrays (TMA) were constructed as previously described^38^. Hematoxylin and eosin (H&E)-stained slides from tissue blocks had been reviewed for adequacy of the representative areas of interest with a high density of tumour cells. The corresponding regions were marked on archival formalin-fixed, paraffin-embedded (FFPE) tissue blocks. The representative areas (2 mm wide and 6 mm long) were extracted, with at least three cores taken from different regions of the tumour, and then vertically planted into the recipient block one by one according to the corresponding location. The planting surface was aggregated on the aggregation instrument. An array was constructed with a maximum of 70 cores and a stomach core was used as an orientation marker.

### Histologic evaluation of TIL

TILs were performed in H&E-stained FFPE sections, which were examined at ×10 and ×40 magnification fields using a conventional light microscope by two pathologists.

Neither pathologist had any knowledge of the clinical information. Intratumoural infiltrating lymphocytes (iTIL) was defined as the percentage of mononuclear cells within the epithelium of the invasive tumour cell nests. Stromal infiltrating lymphocytes (sTIL) was defined as the percentage of tumour stroma containing infiltrating lymphocytes (area occupied by mononuclear cells in tumor stroma/total stromal area). Here, in our study, more than 10% of either stromal or intratumoural TIL were defined as high TIL.

### Immunohistochemical analysis

Three- to 5-μm thick ESCC tissues were consecutively cut, subsequently dewaxed and rehydrated through graded alcohols. Slides were immunohistochemically stained in Roche Ventana Benchmark XT automated slide stainer (Ventana Medical Systems, Roche, France) according to the manufacturer’s instructions. Monoclonal and polyclonal anti-human antibodies were used for identification of CD4+ T cells (anti-CD4, 4B12, Dako), CD8+ T cells (anti-CD8, Ab4055, Abcam), and Foxp3+ T cells (anti-Foxp3, 236 A/E7, Abcam). Quantitative evaluation of CD4+, CD8+ and Foxp3+ T cells was performed in 10 most representative high-power fields (magnification 40×) per tissue section using a Leica DM2000 microscope (Leica Co, Germany). The number of cells with positive staining was counted manually in tumor areas and stromal areas. The results were carried out blindly to the clinical data.

### Statistical analysis

The associations between TIL and clinicopathologic features were analyzed using the chi-square (χ^2^) test. Time-to-event outcomes were defined from date of initially curative resection to date of last follow-up, progression (disease free survival, DFS and disease specific overall survival, OS). Univariate analysis was based on the Cox proportional hazards regression model. A multivariate Cox forward stepwise regression model was used to detect independent predictors of survival. The survival curves were compared using Kaplan-Meier method and log-rank test.

All tests were two sided, and a *P*-value of less than 0.05 was considered as statistical significance. Data were analyzed using SPSS version 21.0 software (SPSS Inc., Chicago, IL, USA).

## Additional Information

**How to cite this article**: Jiang, D. *et al*. Tumour infiltrating lymphocytes correlate with improved survival in patients with esophageal squamous cell carcinoma. *Sci. Rep.* **7**, 44823; doi: 10.1038/srep44823 (2017).

**Publisher's note:** Springer Nature remains neutral with regard to jurisdictional claims in published maps and institutional affiliations.

## Figures and Tables

**Table 1:** Clinicopathological factors and association with TILs in ESCC.

	No.	sTIL (>10%)		*P* value	iTIL (>10%)		*P* value
		Low	High		Low	High	
Sex				0.634			0.761
Female	38	23	15		23	15	
Male	197	111	86		114	83	
Age				0.472			0.904
<60	97	58	39		57	40	
≥60	139	77	62		80	58	
Differentiation				0.377			0.023
well	4	3	1		3	1	
moderate	133	71	62		87	46	
poor	98	60	38		47	51	
Vessel invasion				0.798			0.545
No	189	107	82		112	77	
Yes	46	27	19		25	21	
Nerve invasion				0.498			0.518
No	166	97	69		99	67	
Yes	69	37	32		38	31	
Lymph node metastasis				0.475			0.383
No	111	66	45		68	43	
Yes	124	68	56		69	55	
Site				0.026			0.210
Upper	12	4	8		4	8	
Middle	112	73	39		67	45	
Lower	102	52	50		59	43	
Stage				0.699			0.646
I–II	136	79	57		81	55	
III–IVa	99	55	44		56	43	
Smoking				0.624			0.570
No	134	78	56		80	54	
Yes	100	55	45		56	44	

sTIL, stromal infiltrating lymphocytes; iTIL, intratumoural infiltrating lymphocytes.

**Table 2:** Univariate and multivariate Cox regression analyses of patient and tumour factors with DFS and OS.

	P value	DFS	P value	OS
		HR (95% CI)		HR (95% CI)
**Univariate analysis**				
sTIL (>10%)	0.083	0.743 (0.531–1.040)	0.037	0.697 (0.496–0.979)
iTIL (>10%)	0.508	1.119 (0.802–1.561)	0.616	1.090 (0.777–1.530)
iTIL (>20%)	0.050	0.508 (0.259–0.999)	0.042	0.477 (0.234–0.974)
sCD4 (>10%)	0.741	0.897 (0.472–1.707)	0.490	0.788 (0.401–1.549)
sCD8 (>10%)	0.791	0.953 (0.667–1.361)	0.569	0.900 (0.626–1.294)
iCD8 (>10%)	0.706	0.929 (0.634–1.361)	0.585	0.896 (0.603–1.330)
sFoxp3 (>10%)	0.641	0.717 (0.177–2.900)	0.604	1.447 (0.358–5.854)
iFoxp3 (>10%)	0.478	0.603 (0.149–2.437)	0.239	0.307 (0.043–2.194)
Sex	0.991	0.999 (0.799–1.249)	0.901	1.015 (0.808–1.274)
Age	0.927	0.985 (0.705–1.375)	0.967	0.993 (0.709–1.390)
Grade	0.106	1.300 (0.946–1.788)	0.142	1.273 (0.922–1.757)
Vessel invasion	0.002	1.793 (1.235–2.605)	<0.001	1.996 (1.371–2.906)
Nerve invasion	0.401	1.164 (0.817–1.659)	0.226	1.248 (0.872–1.785)
Lymph node metastasis	<0.001	3.128 (2.178–4.494)	<0.001	3.476 (2.404–5.026)
Stage	<0.001	3.274 (2.330–4.600)	<0.001	3.580 (2.536–5.053)
Site	0.042	0.758 (0.580–0.990)	0.066	0.775 (0.590–1.017)
Smoking	0.369	1.164 (0.836–1.619)	0.178	1.257 (0.901–1.754)
**Multivariate analysis**				
sTIL (>10%)	0.019	0.664 (0.473–0.934)	0.005	0.608 (0.431–0.857)
iTIL (>20%)	0.095	0.561 (0.284–1.106)	0.087	0.534 (0.260–1.095)
Vessel invovlement	0.506	1.142 (0.773–1.687)	0.327	1.217 (0.822–1.803)
Lymph node metastasis	0.010	1.950 (1.169–3.252)	0.004	2.118 (1.265–3.545)
Stage	0.004	2.040 (1.256–3.312)	0.002	2.119 (1.303–3.446)

DFS, disease free survival. OS, disease specific overall survival. HR, hazard ratio. CI, confidence interval. sTIL, stromal infiltrating lymphocytes. iTIL, intratumoural infiltrating lymphocytes. sCD4, CD4+ stromal infiltrating lymphocytes. sCD8, CD8+ stromal infiltrating lymphocytes. iCD8, CD8+ intratumoural infiltrating lymphocytes. sFoxp3, Foxp3+ stromal infiltrating lymphocytes. iFoxp3, Foxp3+ intratumoural infiltrating lymphocytes.

**Table 3:** Univariate and multivariate Cox regression analyses of patient and tumour factors with DFS and OS in Stage III-IVa patients.

	P value	DFS	P value	OS
		HR (95% CI)		HR (95% CI)
**Univariate analysis**				
sTIL (>10%)	0.031	0.615 (0.395–0.957)	0.007	0.544 (0.349–0.850)
iTIL (>10%)	0.620	0.895 (0.577–1.389)	0.557	0.875 (0.561–1.366)
iTIL (>20%)	0.046	0.309 (0.097–0.980)	0.061	0.331 (0.104–1.052)
sCD4 (>10%)	0.521	0.787 (0.379–1.635)	0.399	0.730 (0.351–1.518)
sCD8 (>10%)	0.078	0.654 (0.408–1.048)	0.036	0.599 (0.371–0.968)
iCD8 (>10%)	0.329	0.780 (0.474–1.284)	0.314	0.767 (0.458–1.285)
sFoxp3 (>10%)	0.182	0.260 (0.036–1.878)	0.160	0.241 (0.033–1.749)
Sex	0.336	0.710 (0.354–1.425)	0.449	0.868 (0.600–1.254)
Age	0.409	1.202 (0.777–1.859)	0.340	1.238 (0.799–1.919)
Grade	0.922	0.980 (0.658–1.461)	0.804	0.950 (0.633–1.426)
Vessel invasion	0.596	1.128 (0.723–1.760)	0.366	1.230 (0.786–1.925)
Nerve invasion	0.322	1.249 (0.804–1.939)	0.245	1.300 (0.836–2.021)
Lymph node metastasis	0.101	0.521 (0.239–1.137)	0.208	0.606 (0.278–1.321)
Site	0.011	0.620 (0.429–0.896)	0.013	0.622 (0.427–0.905)
Smoking	0.295	0.792 (0.513–1.224)	0.296	0.791 (0.510–1.228)
**Multivariate analysis**				
sTIL (>10%)	0.081	0.671 (0.428–1.051)	0.032	0.535 (0.303–0.947)
iTIL (>20%)	0.056	0.322 (0.101–1.028)	0.071	0.341 (0.106–1.095)
sCD8 (>10%)			0.785	1.091 (0.584–2.040)
Site	0.038		0.032	
Middle	0.233	0.557 (0.213–1.457)	0.144	0.479 (0.178–1.285)
Lower	0.036	0.359 (0.138–0.934)	0.022	0.311 (0.114–0.848)

DFS, disease free survival. OS, disease specific overall survival. HR, hazard ratio. CI, confidence interval. sTIL, stromal infiltrating lymphocytes. iTIL, intratumoural infiltrating lymphocytes. sCD4, CD4+ stromal infiltrating lymphocytes. sCD8, CD8+ stromal infiltrating lymphocytes. iCD8, CD8+ intratumoural infiltrating lymphocytes. sFoxp3, Foxp3+ stromal infiltrating lymphocytes. iFoxp3, Foxp3+ intratumoural infiltrating lymphocytes.

**Table 4:** Univariate and multivariate Cox regression analyses of patient and tumour factors with DFS and OS in Stage I-II patients.

	P value	DFS	P value	OS
		HR (95% CI)		HR (95% CI)
**Univariate analysis**				
sTIL (>10%)	0.315	0.766 (0.455–1.289)	0.189	0.700 (0.412–1.191)
iTIL (>10%)	0.379	1.258 (0.754–2.097)	0.488	1.225 (0.726–2.067)
iTIL (>20%)	0.624	0.810 (0.348–1.883)	0.460	0.707 (0.282–1.772)
sCD4 (>10%)	0.400	0.546 (0.133–2.235)	0.192	0.268 (0.037–1.938)
sCD8 (>10%)	0.554	1.180 (0.683–2.037)	0.730	1.103 (0.631–1.929)
iCD8 (>10%)	0.898	0.962 (0.529–1.750)	0.817	0.930 (0.501–1.724)
sFoxp3 (>10%)	0.582	1.742 (0.241–12.590)	0.604	1.689 (0.233–12.220)
iFoxp3 (>10%)	0.981	1.018 (0.248–4.173)	0.536	0.535 (0.074–3.871)
Sex	0.466	0.895 (0.663–1.207)	0.369	0.871 (0.645–1.177)
Age	0.729	1.099 (0.646–1.868)	0.592	1.160 (0.674–1.994)
Grade	0.064	1.600 (0.974–2.631)	0.124	1.487 (0.897–2.465)
Vessel invasion	0.681	1.194 (0.513–2.777)	0.544	1.300 (0.558–3.031)
Nerve invasion	0.038	0.435 (0.197–0.956)	0.074	0.487 (0.221–1.074)
Lymph node metastasis	<0.001	2.689 (1.600–4.518)	<0.001	2.970 (1.757–5.021)
Site	0.034	0.646 (0.431–0.968)	0.049	0.660 (0.436–0.998)
Smoking	0.617	1.142 (0.678–1.922)	0.437	1.232 (0.728–2.085)
**Multivariate analysis**				
Nerve involvement	0.168	0.568 (0.255–1.268)		
Lymph node metastasis	0.001	2.382 (1.403–4.044)	<0.001	2.856 (1.685–4.839)
Site	0.010		0.010	
Middle	0.274	1.927 (0.595–6.237)	0.250	1.997 (0.615–6.484)
Lower	0.722	0.799 (0.232–2.749)	0.749	0.816 (0.236–2.823)

DFS, disease free survival. OS, disease specific overall survival. HR, hazard ratio. CI, confidence interval. sTIL, stromal infiltrating lymphocytes; iTIL, intratumoural infiltrating lymphocytes. sCD4, CD4+ stromal infiltrating lymphocytes. sCD8, CD8+ stromal infiltrating lymphocytes; iCD8, CD8+ intratumoural infiltrating lymphocytes. sFoxp3, Foxp3+ stromal infiltrating lymphocytes; iFoxp3, Foxp3+ intratumoural infiltrating lymphocytes.
